# Gigantol Suppresses Cancer Stem Cell-Like Phenotypes in Lung Cancer Cells

**DOI:** 10.1155/2015/836564

**Published:** 2015-08-03

**Authors:** Narumol Bhummaphan, Pithi Chanvorachote

**Affiliations:** ^1^Cell-Based Drug and Health Product Development Research Unit, Faculty of Pharmaceutical Sciences, Chulalongkorn University, Bangkok 10330, Thailand; ^2^Department of Pharmacology and Physiology, Faculty of Pharmaceutical Sciences, Chulalongkorn University, Bangkok 10330, Thailand

## Abstract

As cancer stem cells (CSCs) contribute to malignancy, metastasis, and relapse of cancers, potential of compound in inhibition of CSCs has garnered most attention in the cancer research as well as drug development fields recently. Herein, we have demonstrated for the first time that gigantol, a pure compound isolated from *Dendrobium draconis*, dramatically suppressed stem-like phenotypes of human lung cancer cells. Gigantol at nontoxic concentrations significantly reduced anchorage-independent growth and survival of the cancer cells. Importantly, gigantol significantly reduced the ability of the cancer cells to form tumor spheroids, a critical hallmark of CSCs. Concomitantly, the treatment of the compound was shown to reduce well-known lung CSCs markers, including CD133 and ALDH1A1. Moreover, we revealed that gigantol decreased stemness in the cancer cells by suppressing the activation of protein kinase B (Akt) signal which in turn decreased the cellular levels of pluripotency and self-renewal factors Oct4 and Nanog. In conclusion, gigantol possesses CSCs suppressing activity which may facilitate the development of this compound for therapeutic approaches by targeting CSCs.

## 1. Introduction

Recent researches in the field of cancer have shown that, within the malignant tumor as well as in the blood of advanced stage cancer patients, there are special cancer cells called cancer stem cells (CSCs) [1]. Such CSCs have been shown to reproduce themselves and sustain the tumor [2, 3]. Moreover, researchers accept that these CSCs account for most aggressive behaviors of the disease including chemotherapeutic resistance, metastasis, and cancer relapse [4–6]. The concept that CSCs are a critical factor driving cancer cell aggressiveness and metastasis has led to the intensive investigations of the novel therapeutic strategies as well as drugs targeting the CSCs [4, 5].

CSCs have been shown to maintain their stemness through the sustained level of several transcription factors as well as the stem cell-related signals. In lung cancer, the expression of Nanog and octamer-binding transcription factor 4 (Oct4) was shown to enhance malignancy through the CSCs induction [7, 8]. Oct4 and Nanog and their activation targets are found to be overexpressed in the CSCs in many types of cancers [9–13] and their expressions associate with the pathogenesis, tumor development, and progression of cancers. The function of Nanog was shown to involve the self-renewal property of the stem cells [14, 15]. Likewise, the activation of Oct4 gene or Oct4 transfection has been shown to promote dedifferentiation and CSCs-like phenotypes [16, 17]. Although the defined upstream molecular mechanisms of such stem cell mediators remain underinvestigated, signal of Akt serine/threonine protein kinase has been widely accepted to play an important role in regulating the self-renewal as well as other stem cell-like phenotypes in CSCs [18, 19]. In non-small-cell lung cancer, Akt signaling was reported to involve with the self-renewal of stem-like cells [20–23]. The phosphorylated Akt was shown to phosphorylate the Oct4 which resulted in the increase of tumorigenic potential [24]. Also, Nanog was shown to be a downstream target of the Akt pathway [25, 26].

In line with the previous studies indicating the anticancer [27] and antimigration [27] activities of gigantol, a bibenzyl compound isolated from the Thai orchid,* Dendrobium draconis*, we aimed to investigate its possible effect on inhibition of CSCs phenotypes as well as related molecular signals in lung cancer cells. The findings gained from the present study may encourage the development and further investigation on gigantol for its use for cancer therapeutic approaches.

## 2. Materials and Methods

### 2.1. Chemicals and Antibodies

Gigantol was isolated from* Dendrobium draconis* as previously described, and its purity was determined using HPLC and NMR spectroscopy with more than 95% purity was used in this study [28]. 3-(4,5-Dimethylthiazol-2-yl)-2,5-diphenyltetrazolium bromide (MTT), Hoechst 33342, propidium iodide (PI), bovine serum albumin (BSA), and dimethyl sulfoxide (DMSO) were purchased from Sigma chemical, Inc. (St. Louis, MO). The following were purchased: CD133 (cat. number CA1217) (Cell Applications, San Diego, CA), ALDH1A1 (cat. number SC-22589) (Santa Cruz Biotechnology), total Akt (cat. number 9272) (Cell signaling Technology), phosphorylated Akt (Ser473) (cat. number 4060) (Cell signaling Technology), Oct4 (cat. number 2750) (Cell signaling Technology), Nanog (cat. number 4903) (Cell signaling Technology), *α*-tubulin (cat. number 2144) (Cell signaling Technology), *β*-actin (cat. number SC-130656) (Santa Cruz Biotechnology), peroxidase-labeled secondary antibodies: anti-rabbit IgG (cat. number 7074) (Cell-signaling Technology) or anti-mouse (cat. number 7076) (Cell-signaling Technology), and Perifosine (Cell-signaling Technology).

### 2.2. Cell Culture

Human non-small-cell lung cancer cell lines, NCI-H460, and human keratinocyte HaCaT cells were obtained from the American Type Culture Collection (Manassas, VA). NCI-H460 was cultivated in Roswell Park Memorial Institute (RPMI) 1640 medium supplemented with 10% fetal bovine serum (FBS), 2 mM L-glutamine, and 100 U/mL penicillin and streptomycin. HaCaT cells were cultivated in Dulbecco's Modified Eagle Medium (DMEM) supplemented with 10% fetal bovine serum (FBS), 2 mM L-glutamine, and 100 U/mL penicillin and streptomycin. Cell cultures were maintained in a 37°C humidified incubator with 5% CO_2_. Cells were routinely passaged at preconfluent density using a 0.25% trypsin solution with 0.53 mM EDTA. RPMI 1640 medium, FBS, L-glutamine, penicillin/streptomycin, phosphate-buffered saline (PBS), trypsin, and EDTA were purchased from GIBCO (Grand Island, NY).

### 2.3. Cytotoxicity Assays

For cytotoxicity assays, cells were seeded onto 96-well plates at a density of 1 × 10^4^ cells/well and were allowed to incubate overnight. Cells were then treated with various concentrations of gigantol and analyzed for cell viability using 3-(4,5-dimethylthiazol-2-yl)-2,5-diphenyltetrazolium bromide (MTT) assay according to the manufacturer's protocol. The cytotoxicity index was calculated by dividing the absorbance of the treated cells by that of the control cells [27, 29].

### 2.4. Cell Death Assay

Nuclear costaining with Hoechst 33342 and propidium iodide (PI) was used to determine apoptotic and necrotic cell death. Cells were incubated with 10 *µ*M Hoechst 33342 and 5 *µ*M PI for 30 min at 37°C. They were visualized and imaged under a fluorescence microscope (Olympus 1X51 with DP70) [27, 30].

### 2.5. Anchorage-Independent Growth Assay

Anchorage-independent growth cell growth was determined by soft agar colony-formation assay. Soft agar was prepared by using a 1 : 1 mixture of RPMI 1640 medium containing 10% FBS and 1% agarose. The mixture was allowed to solidify in a 24-well plate to form a bottom layer, after which an upper cellular layer consisting of 3 × 10^3^ cells/mL in the agarose gel with 10% FBS and 0.3% agarose was added. After the upper layer was solidified, RPMI medium containing 10% FBS was added to the system and incubated at 37°C. Colony formation was determined after 2 weeks using a phase-contrast microscope (Olympus 1X51 with DP70). Relative colony number and area were determined by dividing the values of the treated cells by those of the control cells.

### 2.6. Spheroid Formation Assay

Approximately 2.5 × 10^3^ cells/well were seeded onto a 24-well ultralow attachment plate using RPMI serum-free medium. Treated cells were treated every 3 days. Phase-contrast images of formed primary spheroids were taken at day 7 of treatment using a phase-contrast microscope (Olympus 1X51 with DP70). Primary spheroids were resuspended into single cells, and again 2.5 × 10^3^ cells/well were seeded onto a 24-well ultralow attachment plate using RPMI serum-free medium. Secondary spheroids were allowed to form for 30 days [6, 31].

### 2.7. Western Blot Analysis

Cells were incubated on ice for 45 min with lysis buffer containing 20 mM Tris-HCl (pH 7.5), 1% Triton X-100, 150 mM NaCl, 10% glycerol, 1 mM Na_3_VO_4_, 50 mM NaF, 100 mM PMSF, and protease inhibitor mixture from Roche Molecular Biochemicals (Indianapolis, IN). Cell lysates were analyzed for protein content using BCA protein assay kit from Pierce Biotechnology (Rockford, IL). Equal amounts of denatured protein samples (60 *µ*g) were loaded onto 10% SDS-PAGE for CD133 and ALDH1A1 analysis and equal amounts of denatured protein samples (100 *µ*g) were loaded onto 10% SDS-PAGE for Akt, phosphorylated Akt, Oct4, and Nanog analysis before being transferred to 0.45-*µ*m nitrocellulose membranes (Bio-Rad, Hercules, CA). Transferred membranes were blocked with medium (25 mM Tris-HCl (pH 7.5), 125 mM NaCl, and 0.05% Tween 20 (TBST)) containing 5% nonfat dry milk powder for 30 min and incubated overnight with specific primary antibodies against CD133, ALDH1A1, Akt, phosphorylated Akt (Ser473), Oct4, Nanog, *α*-tubulin, and *β*-actin. Membranes were washed three times with TBST and incubated with the following appropriate horseradish peroxidase-labeled secondary antibodies: anti-rabbit IgG or anti-mouse, for 2 h at room temperature. The immune complexes were detected by SuperSignal West Pico chemiluminescent substrate (Pierce Biotechnology) and exposed to film.

### 2.8. Statistical Analysis

All treatments data were normalized to nontreated controls. Data are presented as the means ± SD from at least three independent experiments. Statistical differences were determined using two-way ANOVA and a post hoc test at a significance level of* P* < 0.05.

## 3. Results

### 3.1. Cytotoxicity of Gigantol on Lung Cancer H460 and Normal Keratinocyte HaCaT Cells

**Figure 1:**
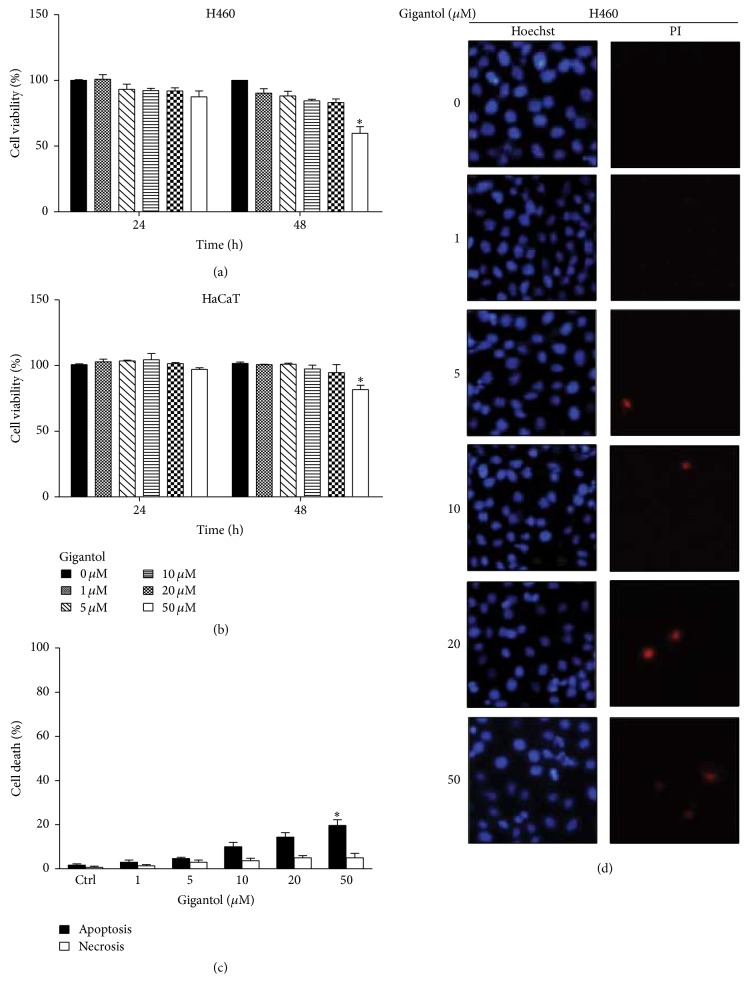
Cytotoxic effect of gigantol on human lung cancer H460 cells. (a) H460 cells and (b) HaCaT cells were treated with various concentrations of gigantol (0–50 *µ*M) for 24 and 48 h. Cell viability was determined by a 3-(4,5-dimethylthiazol-2-yl)-2,5-diphenyltetrazolium bromide (MTT) assay. The viability of untreated cells was represented as 100%. ((c) and (d)) H460 cells were treated with gigantol (0–50 *µ*M) for 48 h. Apoptotic and necrotic cell death were evaluated using Hoechst 33342/PI staining and calculated as a percentage compared with nontreated control cells. All plots are means ± SD (*n* = 3). ^*∗*^ *P* < 0.05 versus nontreated cells.

Previous studies found that CSCs within tumors drive tumor growth and recurrence [2]. To test whether gigantol has an effect on CSCs phenotypes, we first characterized the noncytotoxic concentrations of the tested compound. Human lung cancer cells and normal keratinocyte stem cells were treated with various concentrations of gigantol (0, 1, 5, 10, 20, and 50 *µ*M), and cell viability was determined after 24 and 48 h by MTT viability assay. Gigantol was considered nontoxic at the doses below 20 *µ*M for both the lung cancer H460 and keratinocyte cells (Figures 1(a) and 1(b)). In addition, analysis of the mode of cell death (apoptosis and necrosis) using Hoechst33342/propidium iodide staining assay showed that treatment of the compound at 0–20 *µ*M caused neither apoptosis nor necrosis to H460 cells. The significant increase of apoptosis was only found in the H460 cells treated with 50 *µ*M gigantol (Figures 1(c) and 1(d)).

### 3.2. Gigantol Suppresses CSC-Like Phenotypes

**Figure 2:**
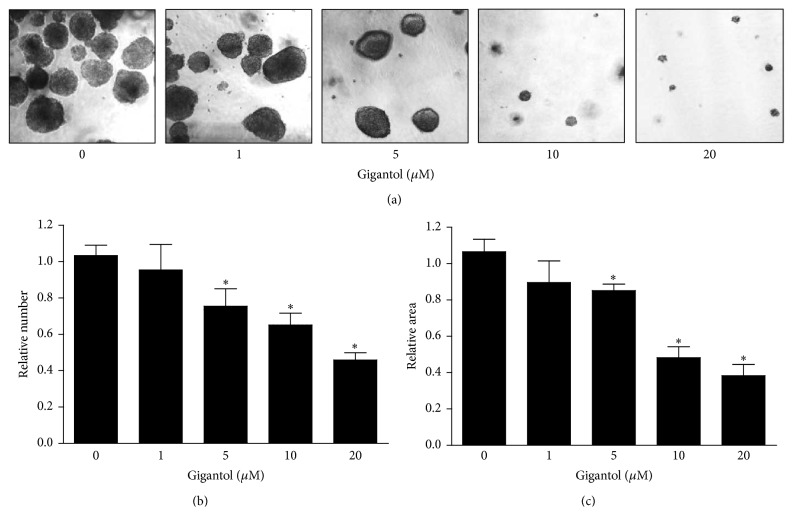
Gigantol inhibits anchorage-independent growth of human lung cancer H460 cells. (a) After being treated with gigantol (0–20 *µ*M) for 48 h, H460 cells were suspended and subjected to anchorage-independent growth assay. (b) Colony number and size were analyzed and calculated as relative values to the control cells. Colony 4x images were captured after day 10. All plots are means ± SD (*n* = 3). ^*∗*^ *P* < 0.05 versus nontreated cells.

As the ability of the cancer cells to form spheroids as well as growth and survival in anchorage-independent condition has been widely accepted as a hallmark of CSCs, we next tested the effect of gigantol on such behaviors. H460 cells were treated with noncytotoxic concentrations of gigantol (0–20 *µ*M) for 48 h, and the cells were subjected to anchorage-independent growth and spheroid formation assays. For anchorage-independent growth, the colony number and colony size were determined and presented as relative values in comparison to those of nontreated control.  Figure 2(a) shows that treatment of the cells with gigantol resulted in the significant decrease of colony number and colony size in a dose-dependent manner. A significant suppression was first detected at 5 *µ*M of gigantol with approximately 30% reduction in terms of colony number and 20% reduction in terms of size.

**Figure 3:**
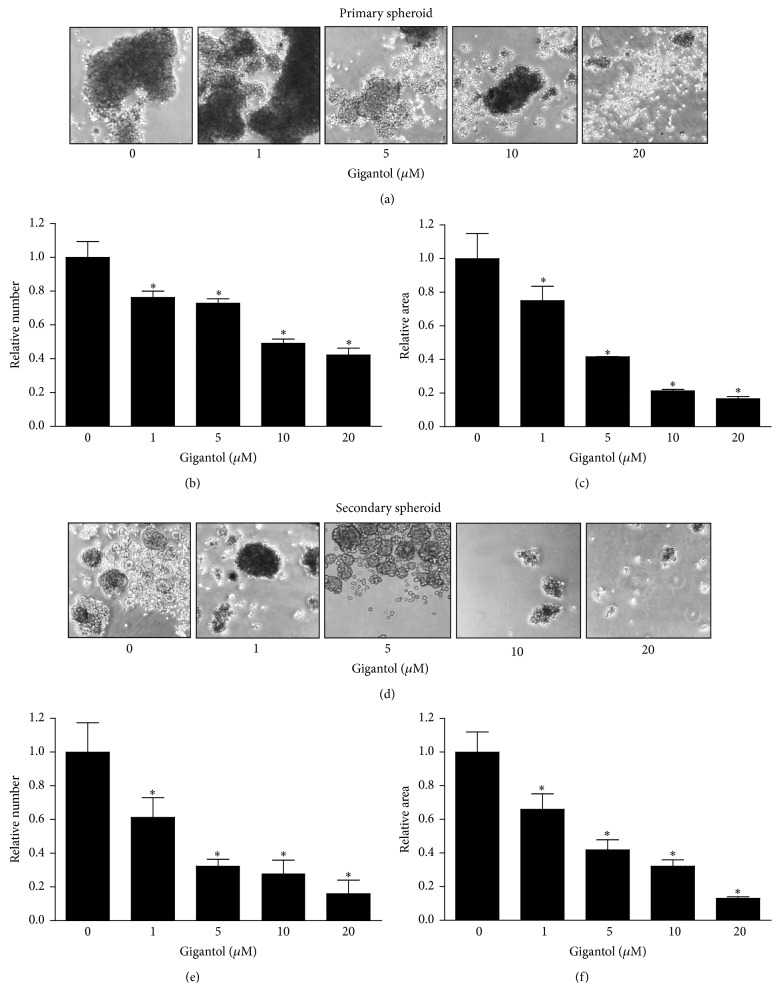
Gigantol suppresses CSC-like phenotypes. (a) After being treated with gigantol (0–20 *µ*M) for 48 h, H460 cells were suspended and subjected to spheroid formation assay. (b) 4x phase-contrast images of primary spheroids at day 7 were captured for treated and nontreated cells. (c) The primary spheroids were resuspended into single cells, and secondary spheroids were allowed to grow for 30 days. (d) 4x phase-contrast images of secondary spheroids at day 30 were presented. All plots are means ± SD (*n* = 3). ^*∗*^ *P* < 0.05 versus nontreated cells.

To confirm the above effect of gigantol on CSCs, the lung cancer cells were similarly treated and subjected to the spheroid formation assay. Cells were pretreated with gigantol for 48 h, detached, resuspended, and seeded at low density onto ultralow attachment plates. The primary spheroids were allowed to form for 7 days (Figure 3(a)). The primary spheroids were then detached and resuspended. The secondary spheroids were allowed to grow for 30 days in RPMI serum-free medium (Figure 3(d)). In the nontreated control cells, the cells have an ability to form aggregates and spheroids in the primary detection. Although the number and size of spheroids were found to be significantly diminished in the secondary spheroids, there are a number of spheroids remaining in such a condition referring to the presence of CSCs in H460 populations. Interestingly, treatment of the cells with nontoxic concentrations of gigantol dramatically reduced both number and size of tumor spheroids (Figure 3), suggesting that the compound has a suppressing effect on the CSCs populations in these cells.

### 3.3. Gigantol Reduces CSC Markers

**Figure 4:**
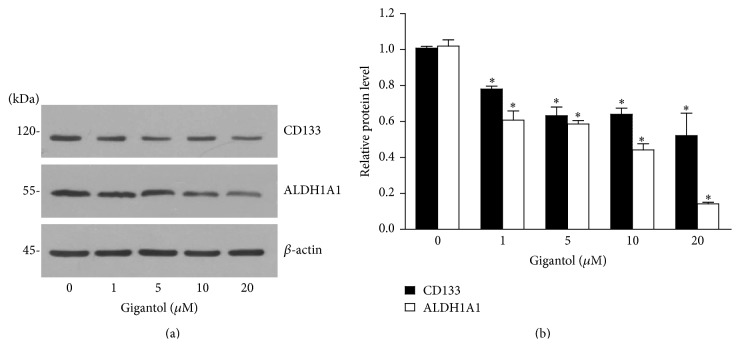
Gigantol reduces CSC markers. (a) After H460 cells were treated with gigantol (0–20 *µ*M) for 48 h, cells were collected, and CSC markers, CD133 and ALDH1A1, were analyzed by Western blotting. The blots were reprobed with *β*-actin to confirm equal loading. (b) Band density was quantified by densitometry, and mean data from independent experiments were normalized to the controls. The bars are means ± SD (*n* = 3). ^*∗*^ *P* < 0.05 versus nontreated cells.

Having shown that gigantol suppressed the CSCs phenotypes in the lung cancer cells, we next confirmed such observation by determining the well-known lung CSC markers. The cells were cultivated in the presence or absence of gigantol for 48 h, and the expression levels of CD133 and ALDH1A1 were determined by Western blotting.  Figure 4 shows that treatment of the cells with gigantol significantly suppressed CD133 and ALDH1A1 expressions in a dose-dependent manner, confirming that gigantol suppresses CSCs phenotypes in lung cancer cells.

### 3.4. Gigantol Suppresses Oct4 and Nanog Reduction through Akt-Dependent Mechanism

**Figure 5:**
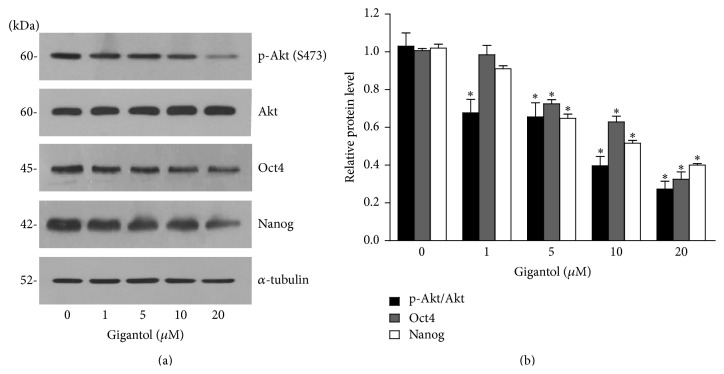
Gigantol suppresses Oct4 and Nanog through Akt-dependent mechanism. (a) After H460 cells were treated with gigantol (0–20 *µ*M) for 48 h, cells were collected, and the cellular levels of self-renewal pluripotency transcription factor, Oct4 and Nanog, were analyzed by Western blotting. The blots were reprobed with *α*-tubulin to confirm equal loading. (b) Signals were quantified by densitometry, and mean data from independent experiments were normalized to the controls. The bars are means ± SD (*n* = 3). ^*∗*^ *P* < 0.05 versus nontreated cells.

**Figure 6:**
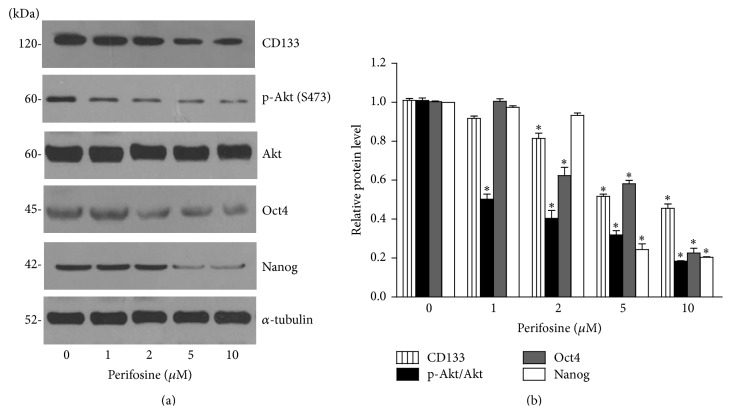
Akt inhibitor perifosine suppresses CD133, Oct4, and Nanog. (a) H460 cells were treated with perifosine (0–10 *µ*M) for 48 h. Cells were collected and CSCs marker CD133 and the cellular levels of self-renewal pluripotency transcription factors, Oct4 and Nanog, were analyzed by Western blotting. The blots were reprobed with *α*-tubulin to confirm equal loading. (b) Signals were quantified by densitometry, and mean data from independent experiments were normalized to the controls. The bars are means ± SD (*n* = 3). ^*∗*^ *P* < 0.05 versus nontreated cells.

The activity of phosphorylated Akt has been shown to link with the proliferation and self-renewal properties of normal and cancer stem cells [12, 24, 32–35]. Evidence has suggested that Akt activity resulted in the increase of cellular levels of self-renewal pluripotency transcription factor Oct4 and Nanog [25, 36, 37]. We further tested whether gigantol suppressed the CSCs through such a pathway. Cells were treated with the nontoxic concentrations of gigantol for 48 h, and phosphorylated Akt, total Akt, Oct4, and Nanog were determined by Western blotting.  Figure 5 shows that the treatment of the cells with gigantol caused decrease of phosphorylated Akt in a dose-dependent manner, whereas total Akt was not altered in comparison to those of nontreated control. Also, its downstream transcription factors including Oct4 and Nanog were found to be significantly reduced following the reduction of phosphorylated Akt. Previous study showed that perifosine (known as Akt inhibitor) reduced the number of mammospheres [38]. To confirm that gigantol regulates Nanog and Oct4 mediated by Akt-dependent mechanism, we used perifosine to study. H460 cells were treated with noncytotoxic concentrations of perifosine (0–10 *µ*M) for 48 h, and the stem cell-regulating proteins were analyzed using Western blot analysis.  Figure 6 shows that treatment of the cells with perifosine significantly reduced phosphorylated Akt with only minimal change of total Akt. Importantly, such an Akt inhibitor significantly suppressed CD133 expression in a dose-dependent manner. Also, the downstream transcription factors including Oct4 and Nanog were found to decrease as a consequence of phosphorylated Akt reduction. Therefore, our results have demonstrated that gigantol possesses the CSCs reducing effect and could be beneficial for the treatment of lung cancer by targeting CSCs.

## 4. Discussion

Lung cancer has been recognized as a major cause of cancer-related death because of high incidence and relapse [39]. Recent studies have shown that the CSCs presenting in the lung cancer may facilitate the malignancy and progression of the disease [7]. Important hallmarks of CSCs are abilities to resist chemotherapeutic drugs, spread, and generate the new tumors [4, 40]. In searching for the potential compounds targeting CSCs, many researchers have focused on the compounds that can suppress stem cell-related pathways. The phosphorylated Akt, a well-known survival and proliferating signal, has been long shown to play an important role in regulating stemness in many cell models [18, 41]. In case of drug development, DC120, a novel Akt inhibitor, was shown to suppress nasopharyngeal carcinoma cancer stem-like cells [42]. Also, the short hairpin RNA of Akt is able to suppress the proliferation and self-renewal of lung cancer stem cells [18]. These data highlight the possibility that compounds inhibiting Akt pathway and related downstream stem cell pathways may benefit the treatment of lung cancer.

We have demonstrated herein for the first time that gigantol, a pure compound isolated form* Dendrobium draconis*, exhibited CSCs suppressing activity in human lung cancer cells. Treatment of the cancer cells with gigantol resulted in the decrease of CSCs indicated by the reduction of cancer cell growth in an anchorage-independent condition as well as the decrease of spheroid formation (Figures 2 and 3). As CD133 and ALDH1A1 have been widely accepted as stem cell marker in lung cancer [6, 43–46], we evaluated the expression of both proteins and found that both proteins were significantly downregulated in gigantol-treated cells. Also, gigantol was shown to suppress stemness through the inhibition of Akt-dependent Oct4 and Nanog reduction (Figure 5). Oct4 and Nanog are the transcription factors frequently found in the stem cells and their functions contribute to the self-renewal and pluripotency of stem cells. Previous studies showed that high expression or ectopic forced expression of Oct4 and Nanog in lung cancer cells transforms the lung cancer cells to CSC-like phenotypes [7, 35, 47, 48]. The high expression level of Nanog in many cancers is also recognized as an indicator of a poor prognosis [49]. Evidence suggested that Oct4 and Nanog are involved in the maintenance of pluripotency and self-renewal in CSCs. Overexpression of Oct4 and Nanog enhanced colony-forming efficiency and promoted the differentiation properties [35, 50]. The knockdown of both transcription factors was reported to decrease proliferation and invasion and reverse the epithelial-mesenchymal transition (EMT) of CSCs [7, 51]. In terms of upstream signaling pathway, Akt has been shown to modulate stem cell homeostasis in the differentiation process of embryonal carcinoma cells (ECCs). Previous studies revealed that phosphorylated Akt plays the critical role in the self-renewal of embryonic stem cells through Oct4 [24, 52]. In addition, phosphorylated Akt is important for the proliferation and maintenance of pluripotency of CSCs as well as sphere formation [53].

**Figure 7:**
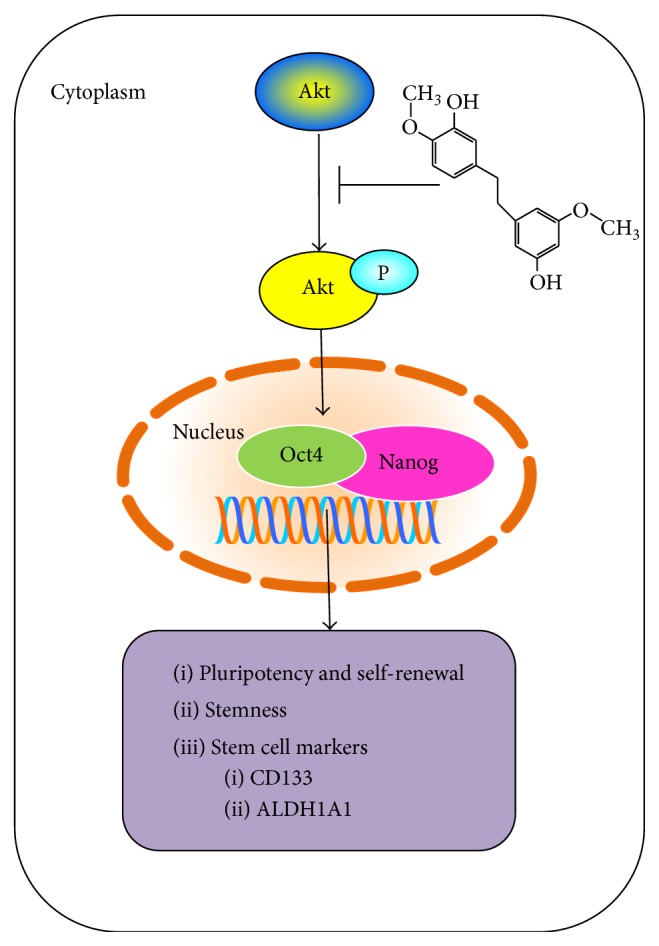
The scheme represents the effect of gigantol on human lung cancer cells. The present study reveals that gigantol has an ability to reduce CSCs markers including CD133 and ALDH1A1 in the cancer cells by suppressing the activation of protein kinase B (Akt) signal which in turn decreased the cellular levels of pluripotency and self-renewal factors Oct4 and Nanog.

In conclusion, we reported a novel finding on the effect of gigantol in suppression of stemness and other CSC-like phenotypes in human lung cancer cells. We have demonstrated that the compound suppresses CSCs features by suppressing the Akt signal leading to the decrease of stem cell factors Oct4 and Nanog (Figure 7). Because CSCs have been tightly linked to the progression of cancer, aggressiveness, and metastasis, the findings of this study could be beneficial to the development of this compound to be useful for cancer therapeutic approaches.
